# Persistence of phlebotomine *Leishmania* vectors in urban sites of Catania (Sicily, Italy)

**DOI:** 10.1186/s13071-014-0560-0

**Published:** 2014-12-09

**Authors:** Oscar Lisi, Vera D’Urso, Valerio Vaccalluzzo, Gioia Bongiorno, Cristina Khoury, Francesco Severini, Trentina Di Muccio, Marina Gramiccia, Luigi Gradoni, Michele Maroli

**Affiliations:** Department of Biological, Geological and Environmental Sciences, Section of Animal Biology “M. La Greca”, University of Catania, Via Androne 81, 95124 Catania, Italy; Unit of Vector-Borne Diseases and International Health, MIPI Department, Istituto Superiore di Sanità, Viale Regina Elena 299, 00161 Rome, Italy

**Keywords:** Catania, Italy, Canine leishmaniasis, Sand fly vectors, *Phlebotomus perniciosus*, Urban sand flies, Phlebotomus, Perniciosus, Leishmania, Sicily

## Abstract

**Background:**

Pioneering research on “Mediterranean Kala-Azar” carried out by Adler and Theodor early in the past century (~1930s) had identified Catania city (Sicily) as a major focus of the disease nowadays known as zoonotic visceral leishmaniasis (VL). Despite the fact that disease in both humans and dogs has continued to be highly prevalent in the Catania province up to the present times, research on *Leishmania* vectors in this urban focus dates back to that distant period. This study aimed to evaluate the persistence and current composition of the sand fly fauna in urban environments of Catania in recent years, 2006 and 2013.

**Methods:**

In 2006 fifty-one suitable collecting sites were identified within 44 sub-units of a grid drawn to include the urban Catania area. In 2013 the survey was restricted to four of the most productive and representative sites resulting from the 2006 survey. In both periods 3 collections per month were performed using standard sticky traps set for 3 days in wall holes/cavities along public roads, from the end of April through December.

**Results:**

43/51 sites (84.3%) were found positive for sand flies. The 2006 collections accounted for a total of 4341 specimens including six species. Among competent *Leishmania* vector species, *P. perniciosus* was the most prevalent (36.5%) being identified in all sand fly-positive sites, with significant abundance in those of the old city centre. Other species of interest were *P. sergenti* (2.5%) and *P. neglectus* (1.5%). The 2013 survey produced 1130 sand flies, of which 39.5% were *P. perniciosus*, 1.6% *P. sergenti* and 0.7% *P. neglectus*. A search for *Leishmania* DNA in a small sample of 72 *P. perniciosus* females revealed 11% infection prevalence.

**Conclusions:**

Our findings from an old urban focus of leishmaniasis demonstrate that phlebotomine sand flies have adapted fairly well to the drastic environmental changes that have occurred in cities of the Western world in the past century and still represent a potential risk for *Leishmania* transmission.

## Background

Leishmaniasis caused by *Leishmania infantum* is a sand fly-transmitted zoonosis resulting in localized cutaneous (CL) or visceral disease (VL) in humans, and viscero-cutaneous disease (CanL) in susceptible dogs that represent the main source of infection. In the past decade the urban pet populations have greatly increased in western Mediterranean areas, resulting in a risk for urban *Leishmania* transmission in cities where suitable conditions for phlebotomine vector breeding are available.

The city of Catania (Sicily, Italy) has long been known as an urban focus of leishmaniasis. Early in the past century (~1930s) the Adler and Theodor’s pioneering investigations on “Mediterranean Kala-Azar” (nowadays known as zoonotic VL) led to the identification of Catania as one of the main VL foci over the whole Mediterranean subregion, along with other southern Italian urban/peri-urban foci such as Naples and Palermo. At that time, the yearly incidence of human VL in Catania was as high as 150-200 cases over a population of some 260.000 inhabitants [1]. Despite the continued high prevalence of the disease in both humans and dogs in the Catania province up to the present times, research on *Leishmania* vectors in this urban focus dates back to the 1930s [2], as no dedicated entomological studies have been carried out thereafter. The above authors reported detailed information on the bionomics and vector competence of the local sand fly fauna, which were captured at sight principally in houses of individuals currently presenting with VL. All species detected were found at high densities in peripheral districts of the city, although they were also recorded in the old city centre although at lower densities. In particular, *Phlebotomus perniciosus* was found irregularly distributed in several urban sites. Even in the same street a house could be found “infested” by this species, whereas neighbouring houses were not. In most instances houses were found infested by other sand fly species such as *Phlebotomus papatasi*, *Phlebotomus neglectus* (= *Phlebotomus major s.l.*), *Phlebotomus sergenti*, and sometimes also by *Sergentomyia minuta* (*=*var. of *P. parroti* Adler & Theodor).

In the present paper we report the results of two entomological surveys performed in 2006 and 2013, aimed at studying the persistence and current composition of *Leishmania* phlebotomine vectors in urban environments of Catania.

## Methods

### Study area

**Figure 1:**
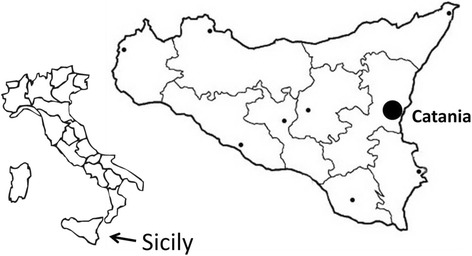
**Geographic area of study.** Sketch map of the Sicily island showing Catania province (large dot) and other chief towns (small dots).

Catania, a port city of the Mediterranean sea (37°30'4"68 N; 15°4'27"12 E) is the second biggest city of Sicily (Figure 1). It lies in the central part of the eastern island coast, overlooking the Ionian Sea at the foot of mount Etna, the highest volcano (3350 m a.s.l.) in Europe. It has a population of about 300,000 inhabitants, while the entire metropolitan territory comprises about 750,000 inhabitants, making Catania one of the most densely populated urban areas of Italy (1662 inhabitants/km^2^). During the past 50 years there has been intense building activity so that the modern town has spread towards the slopes of Etna at the north and to the north-east. The climate is typically Mediterranean; the cold season is usually short and guarantees mild temperatures during the daytime. Summer is long, very hot and usually with low humidity. In districts along the coast the elevated temperatures are partially attenuated by the sea breeze, while in central districts higher temperatures are found all day and night long.

**Figure 2:**
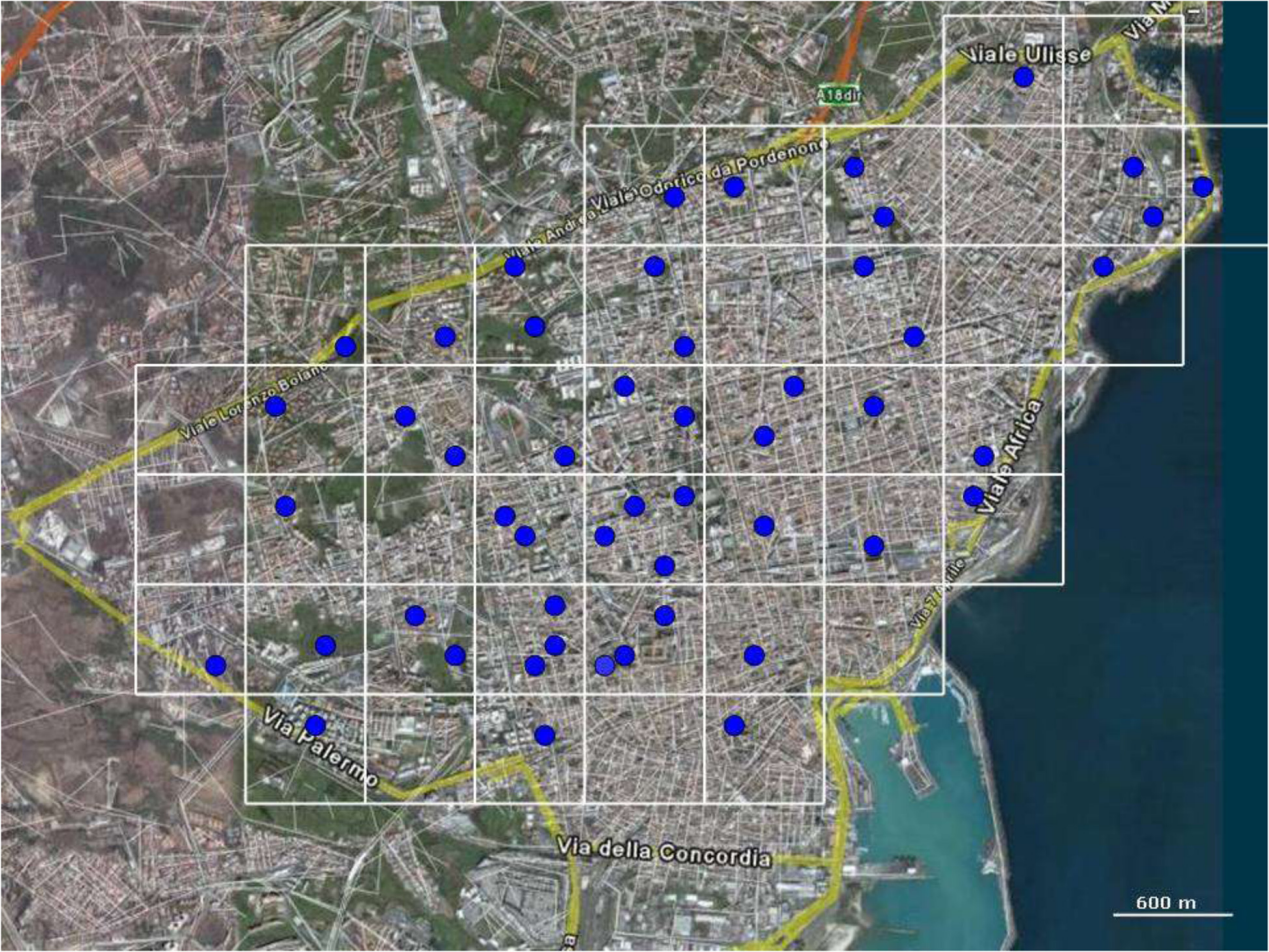
**Aerial view of the city of Catania.** Blue circles represent the sites monitored for sand flies located within a grid of 44 sub-units of 600 m x 600 m each.

The urban Catania territory surveyed in the present study lies between wide avenues at north and north-west, two main streets at south and south-east, and the sea coast to the east (Figure 2). These borders include the majority of the urban areas, with the older districts to the south and the newest ones to the north. Although the area is densely populated, there are green areas such as public gardens and some residual strips of spontaneous vegetation or abandoned fields interspersed with lava rock. Old houses are built of lava stone and lime; they usually have a small courtyard and/or a little private green area, and on their external walls large holes are commonly present originally used for fixing the timbers of the scaffolding while building the house.

### Collecting sites

**Figure 3:**
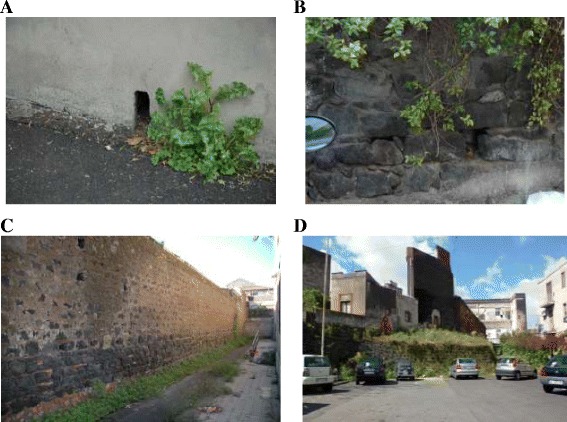
**Four collecting sites monitored in 2006 and 2013. A**, site No. 9 showing a typical hole for water drainage into a retaining wall; **B**, site No. 35, with two holes in a dry wall built with lava stones; **C**, site No. 36, made up of a long drywall with many holes for water drainage; **D**, site No. 37, showing a drywall against an embankment with many holes for water drainage.

The studied area was divided by a grid of 44 sub-units of 600 m x 600 m each (Figure 2). Potential daytime resting sites in public accessible places were selected to monitor the sand fly presence. They consisted of drainage cavities of retaining walls, holes in external old house walls, natural or artificial cavities in lava rock walls, or drain pipes (Figure 3). Only a few cases of indoor resting sites (houses, cellars) were monitored. A total of 51 suitable collecting sites were identified in 34/44 sub-units (1-3 sites by sub-unit). All sites were investigated in 2006, whereas in 2013 the survey was restricted to 4 of the most productive and representative ones.

### Sand fly collection and classification

Standard sticky traps, consisting of 20 cm × 20 cm castor-oiled sheets, were used to monitor the sand fly presence. Traps were set for 3 consecutive nights at intervals of 10 days from the end of April through December. The number of holes/cavities found in each collecting site varied greatly, so that the number of sticky traps used also varied from site to site. On average, 12 sticky traps/site (range 4-28) were left *in situ*. Trapped sand flies were removed with a fine brush soaked in ethanol and sorted by sex. Most of the specimens were cleared in chloro lactophenol and then mounted in permanent preparations for species identification. A small group of females, including some dead specimens recovered from CDC light traps employed in one site for different purposes, was left in ethanol pending identification by dissection and *Leishmania* DNA search. Sand flies were identified at species level by their morphological characteristics according to Theodor [3] and Léger *et al.* [4].

### *Leishmania* infection in sand flies

Presence of *Leishmania* DNA was tested in individual sand fly females. Genomic DNA was extracted using the phenol–chloroform protocol, the DNA pellet was resuspended in 50 μl of sterile water and stored at −20°C pending a nested (n)-PCR assay [5]. *Leishmania* genome amplification was performed using primers for the small subunit (SSU) rDNA [6]. Positive samples yielded a predicted n-PCR product of 358 bp.

## Results

### Urban sand fly fauna

By the end of May 2006, 8/51 sites (15.6%) located in the ancient eastern side of the city were sand-fly negative during three consecutive collections and were therefore excluded from the rest of the study. The remaining 43 sites (see Table 1 for their characteristics) were found sand-fly positive and produced a total of 4339 specimens belonging to *Sergentomyia* (2587 specimens, 59.6%) and *Phlebotomus* genera (1752 specimens, 43.4%). All *Sergentomyia* flies were identified as *S. minuta*. Among the *Phlebotomus* specimens, *P. perniciosus* was the most prevalent and abundant species (1577/1752 specimens, 90.01%), followed by *P. sergenti* (6.16%), *P. neglectus* (3.65%), *P. perfiliewi* (0.12%) and *P. mascittii* (0.06%)*. P. perniciosus* was collected in all sand-fly positive sites, with a cumulative seasonal number of specimens/site ranging 1-210, for an average of 36. Other species were much less spread and/or abundant; *P. sergenti* was collected in 10/43 sites, with a total of 108 specimens, and *P. neglectus* in 6/43 sites, with 64 specimens. *P. mascittii* and *P. perfiliewi* were found to be very rare, being collected in 1 and 2 sites only, respectively. In one site (no. 36, Figure 3C) the sand fly fauna was represented by 5/6 species identified in the study: *P. perniciosus*, *P. sergenti, P. neglectus, P. perfiliewi* and *S. minuta*. This site, located within an ancient degraded district, consisted of a retaining wall in a school courtyard. Sites nos. 35 and 37 (Figure 3B-D) were those producing the majority of *P. sergenti* specimens (92/108, 85.2%) and *P. neglectus* (35/64, 54.7%), respectively.

**Table 1:** **Identification and characteristics of collecting sites found positive for sand flies in Catania city**

**Site no.**	**Street/Square**	**Latitude; Longitude**	**Altitude (m a.s.l.)**	**Vegetation**	**Potential mammal hosts identified** ^**1,2**^
1	Carlo Forlanini	37°30’37”N; 15°04’22”E	66	**-**	Dogs, cats, rodents, men
2	Valdisavoia	37°31’05”N; 15°04’09”E	79	**++**	Dogs, cats, men
3	Chiuse lunghe (North)	37°31’20”N; 15°04’23”E	106	**+**	Dogs, cats, rodents, men
4	Chiuse lunghe (South)	37°31’09”N; 15°04’30”E	66	**++**	Dogs, cats, rabbits, rodents, men
5	Largo Taormina	37°31’02”N; 15°04’41”E	55	**+**	Dogs, cats, men
7	Al Tondo Gioieni	37°31’37”N; 15°05’01”E	90	**-**	Dogs, cats, rodents, men
8	Gustavo Vagliasindi	37°31’28”N; 15°05’42”E	52	**+**	Dogs, cats, rodents, men
9	Vittorio Veneto	37°31’24”N; 15°05’46”E	48	**++**	Dogs, cats, men
10	Sassari	37°31’18”N; 15°05’37”E	46	**++**	Dogs, cats, rodents, men
11	Gabriele D’Annunzio	37°31’01”N; 15°05’23”E	44	**+**	Dogs, cats, men
12	Sabato Martelli Castaldi	37°30’54”N; 15°03’56”E	74	**+++**	Dogs, cats, rabbit, sheep, men
13	Masaniello	37°30’15”N; 15°04’03”E	67	**+++**	Dogs, rodents, men
14	Santa Maria della Catena	37°30’13”N; 15°04’08”E	59	**+**	Dogs, cats, rodents, men
15	Amedeo Duca d’Aosta	37°30’18”N; 15°03’38”E	77	**+++**	Dogs, cats, rabbits, rodents, men
16	Palermo	37°29’57”N; 15°03’35”E	53	**-**	Dogs, cats, rodents, men
17	Fratelli Bandiera	37°30’06”N; 15°03’09”E	67	**-**	Dogs, cats, rodents, men
18	Livenza	37°30’43”N; 15°03’31”E	108	**++**	Dogs, rodents, men
19	Giuseppe Ballo	37°30’59”N; 15°03’30”E	114	**++**	Dogs, cats, rodents, men
20	Antonio Merlino	37°31’06”N; 15°03’40”E	98	**++**	Dogs, cats, rodents, men
21	Edmondo De Amicis	37°31’51”N; 15°06’20”E	38	**+**	Dogs, cats, rodents, men
22	Acireale	37°31’32”N; 15°06’36”E	30	**+**	Dogs, cats, rodents, men
23	Plutone	37°31’26”N; 15°06’41”E	25	**-**	Dogs, cats, rodents, men
24	Derna	37°31’28”N; 15°06’45”E	24	**+**	Dogs, cats, rodents, men
26	Borgo	37°31’03”N; 15°04’55”E	51	**+**	Dogs, cats, rodents, men
27	Etnea	37°31’22”N; 15°04’56”E	80	**-**	Dogs, cats, men
28	Guglielmo Oberdan	37°30’53”N; 15°05’20”E	36	**+**	Dogs, cats, rodents, men
29	Ramondetta	37°30’56”N; 15°05’38”E	33	**-**	Dogs, cats, rodents, men
30	Sabotino	37°30’40”N; 15°05’59”E	28	**-**	Dogs, cats, rodents, men
31	Raffineria	37°30’36”N; 15°06’01”E	22	**-**	Dogs, cats, rodents, men
33	Bambino	37°30’23”N; 15°04’53”E	50	**-**	Dogs, cats, rodents, men
34	Osservatorio	37°30’19”N; 15°04’46”E	52	**-**	Dogs, cats, rodents, men
35	Fiorentino	37°30’29”N; 15°04’42”E	46	**+**	Dogs, cats, rodents,men
36	Zammataro	37°29’49”N; 15°04’24”E	32	**-**	Rodents, cats, men
37	Ammiraglio Toscano	37°30’48”N; 15°04’10”E	73	**+**	Dogs, cats, rodents, men
38	Misterbianco	37°30’23”N; 15°05’47”E	23	**-**	Dogs, cats, rodents, men
39	Antonino Longo	37°30’57”N; 15°04’59”E	45	**++**	Rodents, cats, men
44	Plebiscito	37°30’11”N; 15°04’38”E	49	**+**	Rodents, cats, men
45	Etnea	37°30’38”N; 15°04’58”E	39	**++**	Dogs, cats, rodents, men
46	Salvatore Citelli	37°30’32”N; 15°04’22”E	60	**+**	Cats, rodents, men
47	Mario Rapisardi	37°30’42”N; 15°04’36”E	52	**-**	Dogs, cats, men
49	Vittorio Emanuele	37°30’09”N; 15°05’08”E	17	**+**	Cats, rodents, men
50	Androne	37°30’38”N; 15°04’45”E	49	**-**	Rodents, men
51	Dante	37°30’13”N; 15°04’42”E	46	**-**	Cats, rodents, men

(-) Absent or rare; (+) moderate; (++) abundant; (+++) very abundant.

^1^Presence of lizards and geckos should also be considered as regards *Sergentomyia* hosts.

^2^The most common rodents in the inspected habitats were *Rattus norvegicus*, *Rattus rattus* and *Mus musculus*.

The 2013 survey, performed in collecting sites nos. 9, 35, 36 and 37, produced 1130 sand flies of which 658 were *S. minuta* (58.2%). The identification of *Phlebotomus* specimens confirmed *P. perniciosus* (94.5%), *P. sergenti* (3.8%) and *P. neglectus* (1.7%). Table 2 compares prevalences of *Phlebotomus* species collected in the 4 sites monitored in both 2006 and 2013.

**Table 2:** **Prevalence of** ***Phlebotomus*** **species collected by sticky traps in 4 urban sites of Catania city (Sicily, Italy) in 2006 and 2013**

**Site no.**	**Specimens (MM)**	***P. perniciosus*** **(MM)**	***P. neglectus*** **(MM)**	***P. sergenti*** **(MM)**
**2006**				
9*	89 (75)	89 (75)	0	0
35	247 (228)	210 (197)	35 (29)	2 (2)
36**	84 (70)	79 (68)	2 (1)	3 (1)
37	202 (185)	105 (101)	5 (5)	92 (79)
Total	622 (558)	483 (441)	42 (35)	97 (82)
**%**		**77.65**	**6.75**	**15.59**
**2013**				
9	305 (267)	303 (267)	0	2 (0)
35	85 (81)	75 (71)	8 (8)	2 (2)
36	9 (8)	9 (8)	0	0
37	73 (66)	59 (55)	0	14 (11)
Total	472 (422)	446 (401)	8 (8)	18 (13)
**%**		**94.49**	**1.69**	**3.81**

(MM = males); *one male of *P. mascittii* and **one female of *P. perfiliewi* were also collected.

### Seasonal dynamics

**Figure 4:**
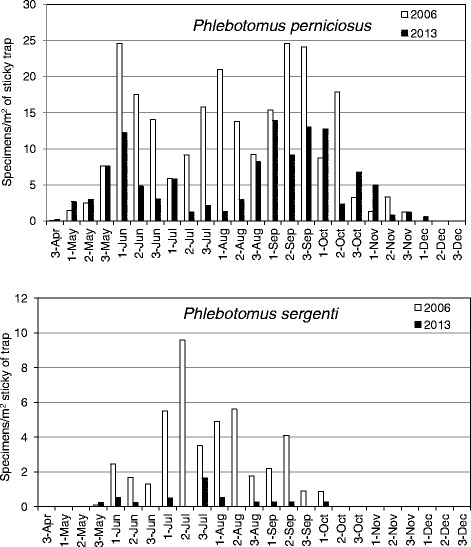
**Seasonal dynamics of** ***P. perniciosus*** **and** ***P. sergenti*** **in 4 urban sites of Catania in 2006 and 2013.** Legend of the x-axis refers to the 3 collections per month during which the sticky traps were left *in situ* for three consecutive nights.

In the 2006 survey, 4/6 species recorded (*P. perniciosus*, *P. sergenti*, *P. neglectus* and *S. minuta*) were collected altogether starting from the last third of May. *P. perniciosus,* already appeared by the last third of April, was not collected by the end of November (in 2013 this species disappeared after the first third of December). *P. sergenti* showed the shortest activity season being already absent after the first third of October. Figure 4 shows the seasonal dynamics of *P. perniciosus* and *P. sergenti* recorded in the 4 sites monitored by both surveys. The comparison in sand fly density (expressed as no. of specimens/m^2^ of sticky trap) revealed differences between the years, being lower for both species in 2013. In 2006 *P. perniciosus* showed 3 density peaks of 21-24 specimens/m^2^ each in June, August and September, whereas in 2013 there were 2 peaks of 12-14 specimens/m^2^ each in June and September/October. In both 2006 and 2013 *P. sergenti* was shown to increase in density between June and September, however, the relatively low number of specimens could only permit identification of a clear density peak in July 2006. The rarity of *P. perfiliewi* and *P. mascittii* makes it difficult to determine their seasonal dynamics.

### *Leishmania* infections

During the 2013 survey, 72 females of *P. perniciosus* collected from the 4 sites were analysed by n-PCR. Eight specimens (11%) resulted positive for genomic *Leishmania* DNA. Positive sites (nos. 9 and 37) were those where the number of collected *P. perniciosus* females was higher: 36 (with 5 positive specimens) and 30 (with 3 positive specimens), respectively. *Leishmania* infections were detected in specimens collected from June through October.

## Discussion

Our study represents the first phlebotomine vector survey in the urban VL focus of Catania some 80 years after the investigations by Alder and Theodor [2]. The 2006 study demonstrated that 6 species of sand flies, of which 4 are proven vectors of human leishmaniases, are endemic in the city. In general, higher sand fly abundance was found in sites of northern and western urban areas where spontaneous or nearly abandoned green areas are commonly present. Lower sand fly abundance was recorded in the eastern sites located in the newest and non-degraded districts of the town.

Our research highlighted the presence of both sand fly genera endemic in the Old World.

All *Sergentomyia* sand flies identified were *S. minuta*. This very common Mediterranean species is known to feed mainly on cold-blooded vertebrates (e.g. lizards and geckos) and it is a proven vector of reptilian Trypanosomatidae species non-pathogenic to humans. In a survey carried out in central and southern Italy in 1986, *L. tarentolae* (syn: *Sauroleishmania tarentolae*) was isolated and typed from *S. minuta*, providing the first proof of these long-suspected parasite-vector associations for mainland Italy [7]. Recently, it has been reported that *S. minuta* can also be a mammophilic species, thus questioning the prevailing opinion that leishmaniases are only transmitted by *Phlebotomus* species in the Old World [8]. However, a recent experimental study on the susceptibility of colonized *S. schwetzi* to *L. donovani*, *L. infantum* and *L. major* suggests that *Sergentomyia* is refractory to human *Leishmania* species [9].

Among *Phlebotomus* species, *P. perniciosus, P. neglectus* and *P. perfiliewi*, all members of the *Larroussius* subgenus, are proven vectors of *L. infantum* [10]. *P. perniciosus* was the most abundant vector species and, being also ubiquitous, can play a major role in the transmission of urban leishmaniasis in the studied area. The high prevalence of natural *Leishmania* infections recorded by molecular methods in a small sample of this species (11%) supports this hypothesis. In addition, the presence of this species in urban environments can be an additional risk to human health because of the proven role in the transmission of Toscana virus (TOSV) (Phlebovirus, Buniaviridae) associated with human cases of meningitis and meningoencephalitis [11]. In Sicily, TOSV has been detected from an American tourist who travelled to this island [12]; moreover TOSV specific antibodies have been documented by recent seroprevalence surveys [13-15]. *P. neglectus* was found rarely in most parts of Catania and was relatively common in only two sites. *P. perfiliewi* was found extremely rarely, being identified in only 2 specimens.

*P. sergenti* was found irregularly distributed, with a ‘hot-spot’ detected in one site (no. 37). This species, a proven vector of CL caused by *L. tropica*, is recorded in many countries of the Mediterranean basin including western Europe and northern Africa. Its migration from Central Asia and subsequent colonization of the Mediterranean region has been hypothesized [16]. In Italy, *P. sergenti* species had been reported in the past only from a few places of Catania area [2,17]. More recently *P. sergenti* was found to be more widespread than originally thought, however, its presence in Italy seems to be restricted to the eastern part of Sicily [18-21] with no evidence of expansion to the mainland. The limited spread and abundance of *P. sergenti* in our survey do not allow us to draw any prediction on the role that the species could play in the transmission of leishmaniasis in the urban area of Catania. However, its presence in Sicily is of particular importance. The island lies in the main immigration route of the “Mediterranean boat people”, migrants who fled African and Middle East countries, most of which are endemic for *L. tropica*, because of civil conflicts and/or poverty. Moreover, Catania is located close to the Sigonella NATO military base, where every year soldiers from all over the world (including *L. tropica*-endemic Middle East countries) pass through.

The finding of one specimen of *P. mascittii* represents the first record of the species in Sicily. It was described for the first time in Rome, Italy, in 1908 [22], and then in other countries of the Mediterranean basin, from Spain to Turkey [23], where it has always been recorded at very low densities. In northern Europe *P. mascittii* was recorded in Germany, Switzerland and Austria where this species is suspected, but not incriminated, to play a role in the transmission of *L. infantum* [24,25]. Finally, the apparent absence of *P. papatasi* in our collections is noteworthy if compared with records by Adler & Theodor [2]. A possible explanation could be that characteristics of monitored sites and/or the collection method employed were unsuitable to catch this species. This is supported by the observation that during the sand fly season in 2013 we caught *P. papatasi* at high densities (>200 specimens) using hand aspirators inside a hen-house in a village not far from the Catania city centre.

**Figure 5:**
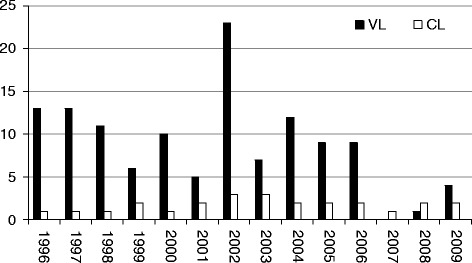
**VL and CL cases diagnosed in Catania province and reported to the Ministry of Health in the period 1996-2009 [** 26 **].**

We have demonstrated the ubiquitous persistence of *P. perniciosus*, the most competent vector of *L. infantum* in Western Mediterranean, in the urban area of Catania some 80 years after the investigations by Adler and Theodor [2]. The question of whether this urban focus is still active as before, or whether there is only a limited risk for *Leishmania* transmission, is important. In Italy, VL and CL cases are subjected to mandatory reporting to the Ministry of Health. However, from the scanty information available, and also because of the typically long incubation period of the diseases, it is unclear whether there were recent human leishmaniasis infections acquired in Catania city. From a retrospective analysis of notifications [26], in the whole Catania province a total of 123 VL cases (average, 8.7/year) and 25 CL cases (average, 1.7/year) were diagnosed in the period 1996-2009. Figure 5 shows the trend of VL and CL cases occurring during that period. It should be noted that CL is often unreported owing to its benign nature. As regards infections in dogs, in recent years only one prevalence study on CanL has been carried out in the Catania area [27]. Clinical evaluation and serological testing were performed on 50 dogs residing with U.S. personnel assigned to Naval Air Station Sigonella, which is far from Catania less than 30 km. Results indicated high exposure to *Leishmania* in the surveyed population, 60% of the tested dogs having elevated anti-*Leishmania* antibody levels. The rate of subclinical infections was 75% among seropositive dogs. Further data on CanL in the Catania province also are those resulting from the *Leishmania* serological testing performed by the Istituto Zooprofilattico Sperimentale (IZS) of Sicily for diagnostic purposes. From a retrospective analysis of the IZS database it appears that a total of 726 dogs were diagnosed as high-titre IFAT positive in Catania province during 2006-2010, of which 120 (16.5%) were dogs residing in the Catania municipality. Our findings on *Leishmania* infections harboured by *P. perniciosus* collected in urban sites would reinforce the hypothesis of a persisting elevated circulation of the parasite in the city of Catania due to the presence of infectious canines and, possibly, infectious cats [28,29].

## Conclusions

The finding of competent vectors in urban areas denotes a potential risk for leishmaniasis transmission. In comparison to the past, when donkey stables, chicken coops and rabbit hutches were widespread in the ancient parts of Catania, suitable hosts on which the phlebotomine vectors can feed are now limited, being prevalently restricted to pets and humans, besides synanthropic rodents (Table 1). Our study has shown that almost all phlebotomine species recorded in the past seem to have fairly adapted to drastic environmental changes occurring in Catania, as in any big city of southern Europe, during the past century. In fact, sand-fly positive resting sites have been identified along urban streets where the intense car traffic produces large amounts of exhaust gases, which apparently did not affect these fragile insects much. Furthermore, given the short flight range of sand flies, we must assume that they also managed to find suitable breeding places in small restricted areas despite the intense housing construction. Presence of suitable vectors and the demonstration of parasite circulation, however, does not necessarily imply the occurrence of human clinical cases of leishmaniasis. The dramatic improvement in the economy and welfare of the Sicilian community after the 2^nd^ World War has probably had a great impact on the human susceptibility to clinical leishmaniasis, which is prevalently a disease of poverty and malnutrition [30]. Thus, it could be interesting to perform investigations on the prevalence of asymptomatic *Leishmania* infections [31] among the resident population of Catania to evaluate the intensity of urban reservoir-to-man transmission.
